# Use of machine learning models to predict mortality in dialysis patients

**DOI:** 10.3389/fpubh.2025.1683285

**Published:** 2025-12-05

**Authors:** Junmin Huang, Lu Chen, Hongying Luo, Junhao Song, Ziqian Bi, Keyu Chen, Xin Liang Chia, Ming Liu, Tianyang Wang, Benji Peng, Zihan Wei, Zhiqing Huang, Zhihang Li, Xincheng Liu, Hongjiu Zhou, Weihuang Zhang, Wen Wen, Mianna Luo, Shujun Wang, Huafeng Liu, Chunjie Tian, Jibin Guan, Joe Yeong, Yongzhi Xu, Peng Wang, Junfeng Hao

**Affiliations:** 1 Guangdong Provincial Key Laboratory of Autophagy and Major Chronic Non-communicable Diseases, Key Laboratory of Prevention and Management of Chronic Kidney Disease of Zhanjiang City, Department of Nephrology, National Clinical Key Specialty Construction Program (2023), Institute of Nephrology, Affiliated Hospital of Guangdong Medical University, Zhanjiang, China; 2 AI Agent Lab, Vokram Group, London, United Kingdom; 3 Purdue University, West Lafayette, IN, United States; 4 AppCubic, Atlanta, GA, United States; 5 Department of Otorhinolaryngology, Affiliated Hospital of Guangdong Medical University, Zhanjiang, China; 6 Masonic Cancer Center, University of Minnesota, Minneapolis, MN, United States; 7 Division of Pathology, Singapore General Hospital, Singapore, Singapore

**Keywords:** maintenance hemodialysis, mortality prediction, machine learning, gradient boosting, risk stratification

## Abstract

**Background:**

Mortality among maintenance hemodialysis patients remains high, and traditional statistical models often fail to capture complex clinical relationships. This study aimed to systematically develop, compare, and validate 19 machine learning algorithms for predicting all-cause mortality in maintenance hemodialysis patients.

**Methods:**

This retrospective study included data from 538 maintenance hemodialysis patients (2018.1–2023.12), with 70% used for training and 30% for testing. Each model underwent hyperparameter optimization based on three performance metrics (accuracy, F1-score, and ROC Area Under the Curve [AUC]) to evaluate the impact of different clinical priorities.

**Results:**

Gradient boosting models demonstrated consistent superiority, with performance outcomes highly sensitive to the selected optimization target. XGBoost optimized for accuracy achieved an F1 score of 0.683 and a ROC AUC of 0.899. AdaBoost optimized for F1 score attained the highest ROC AUC of 0.903 and an F1 score of 0.682. AdaBoost also demonstrated robust performance across optimization strategies, suggesting its suitability for clinical implementation where balanced risk prediction is essential.

**Conclusion:**

A systematic ML framework can yield tailored, high-performing models for mortality risk stratification in maintenance hemodialysis patients, with significant potential to enhance identification and management of high-risk individuals in clinical practice.

**Clinical trial number Registry:**

Chinese Clinica Trial Registry (ChiCTR), TRN:ChiCTR2500103960, Registration date: 9 June 2025.

## 1 Introduction

The mortality rate among patients undergoing hemodialysis is higher than that of patients receiving peritoneal dialysis and the general population. Both short-term survival during the dialysis induction period and long-term survival outcomes remain unsatisfactory (1). It has been reported that the five-year survival rate is less than 50%, primarily due to the high incidence of cardiovascular complications and infectious events in this patient population (2). Despite these challenges, hemodialysis remains the preferred treatment modality for patients with end-stage renal disease (ESRD) according to current clinical guidelines (3). A critical research focus lies in identifying existing clinical variables to construct predictive models for stratifying and preventing high-risk patients.

Classical statistical analyzes have identified several variables that are effective in predicting the mortality rate of hemodialysis patients (4, 5); however, inconsistencies exist between different studies. Few studies have performed risk prediction for multicenter populations, with most models developed based on single-center data. Some scholars argue that such single-center models may better reflect clinical practice. In our previous research at this center, traditional models demonstrated a certain discriminatory ability to predict mortality. However, classical survival analysis methods, such as Cox proportional hazards regression and logistic regression, rely on the assumption of a linear relationship between variables and outcomes (6), which limits their ability to capture complex nonlinear interactions between clinical variables and death outcomes. An increasing number of studies have highlighted U-shaped associations (7, 8), exponential relationships, or threshold effects between specific variables and mortality.

Machine learning (ML) algorithms, intense neural networks, have shown superior predictive performance in stratifying the prognosis of hemodialysis (9). Recent studies have developed multiple machine learning-based survival prediction models for both short-term mortality and long-term survival in maintenance hemodialysis (MHD) patients (10). In addition, machine learning excels at identifying feature variables that are difficult to detect using traditional models (11). However, the clinical application of these algorithms requires clarification of their supportive role rather than decision-making. In addition, there is currently no consensus on optimal models to predict the mortality of hemodialysis patients, necessitating diverse classifier architectures and refined data stratification for model construction. This study aims to provide a more complete risk prediction by fully using patient-specific information through a machine learning framework.

## 2 Methods

### 2.1 Patient population and medical data

This study analyzed data from the Affiliated Hospital of Guangdong Medical University collected between 2018.1 and 2023.12. The primary endpoint was all-cause mortality at 3 years. Inclusion criteria required that patients undergo dialysis at least twice weekly, with each session lasting no < 3 h. A total of 538 patients met these criteria and were included in the study. All participants were aged 18 years or older. The study was conducted in accordance with the Declaration of Helsinki and other international ethical guidelines, and was reviewed and approved by the Ethics Committee of the Affiliated Hospital of Guangdong Medical University.

General information includes age, gender, BMI and comorbidities, such as diabetes, hypertension, cardiovascular diseases, etc. Variables collected at the start of the study after maintaining HD for at least 3 months include hemoglobin, platelets, ferritin, creatinine, albumin, blood uric acid, total cholesterol, triglycerides, C-reactive protein, phosphorus, calcium, potassium, parathyroid hormone, β_2_-microglobulin, etc. The evaluated HD parameters are blood pressure before and after dialysis, residual urine volume, type of HD vascular access, anticoagulant, and dialysis dose expressed as sKt/V urea. The urea nitrogen samples for sKt/V are from venous blood before and after dialysis. The above constitutes the baseline data for the study.

### 2.2 Data processing and feature screening

The primary outcome for this predictive modeling study was all-cause mortality within 3 years, which was coded as a binary variable indicating the patient's survival status at the conclusion of the study's follow-up period. The predictor variables, or features, used for model training were derived from the baseline data collected for each patient. These features were categorized into continuous and categorical types for the analysis.

Mean/median imputation is deliberately avoided in the presence of missing data, since these methods may introduce artificial bias or dilute clinically meaningful signals. Instead, missing data were handled through the following steps (Table 1).

- **Categorical variables** included gender, the type of hemodialysis vascular access, the anticoagulant used, and the presence or absence of key comorbidities (diabetes, hypertension, cardiovascular diseases). These were treated as binary or multiclass categorical inputs.
- **Continuous variables** encompassed a wide range of baseline measurements. These included demographic data (age at study entry, BMI), key laboratory values (e.g., hemoglobin, albumin, C-reactive protein, β_2_-microglobulin), and hemodialysis-specific parameters such as pre-dialysis blood pressure, residual urine volume, and the dialysis dose, quantified by sKt/V.

**Table 1:** Strategy for handling missing data.

**Step**	**Description**
Missingness mask	For each feature, the locations of missing values were first recorded.
Column-wise normalization	Min-Max scaling was applied only to observed values in each feature, ensuring valid ranges were preserved without distorting distributions.
Sentinel encoding	Missing entries were replaced with a constant value of −1, which lies outside the normalized [0, 1] range. This design allows models to recognize and learn from missingness explicitly.
Model compatibility	Tree-based models (e.g., XGBoost, LightGBM) and neural networks were trained with this sentinel encoding. Empirically, models leveraged the missingness signal without performance degradation.

We used both expert knowledge and automated techniques to sifted through a large number of data (Table 2). The influence of irrelevant predictors was further mitigated through the use of the AutoGluon framework.

**Table 2:** Overview of feature selection methodology.

**Step**	**Description**
Domain knowledge filtering	Clinical experts prioritized features known to be relevant for intracerebral hemorrhage (e.g., baseline hematoma volume, midline shift, anticoagulant use, comorbidities).
Univariate filtering	Features with no variability or extremely low variance were removed.
Correlation analysis	Highly correlated features (*r* > 0.9) were reduced to avoid redundancy.
Ablation study	We systematically removed groups of features (see Supplementary **ablation.xlsx**) and compared performance to quantify their contribution.
Model-based importance	Tree-based models (Random Forest, XGBoost) and SHAP analysis were applied to rank features by predictive contribution. Only top-ranked features were retained.

### 2.3 Machine learning modeling

We separated the data into a subset 70% to train the models and the rest 30% to test the models. Hyperparameter optimization for all models was conducted using 5-fold cross-validation in the training set, the left-over test set reserved exclusively for the final evaluation to ensure unbiased performance estimates. To address the class imbalance inherent in this dataset (approximately 23% mortality rate), we systematically evaluated multiple strategies including the Synthetic Minority Oversampling Technique (SMOTE), Adaptive Synthetic Sampling (ADASYN), random undersampling, and class weighting. Comparative analysis via cross-validation revealed that class weighting provided optimal predictive performance without introducing synthetic samples, thereby preserving data integrity while appropriately penalizing misclassification of the minority class. Consequently, class weighting was adopted as the standard approach in all models.

To comprehensively evaluate the potential of machine learning for mortality prediction in this cohort, we implemented and compared a diverse array of 19 supervised learning algorithms. These models were grouped into four distinct families based on their underlying principles: (1) Baseline and Linear Models, (2) Tree-Based Ensemble Models, (3) Other Kernel-Based and Probabilistic Models, and (4) Neural Network-Based Models.

The hyperparameter configurations used in our experiments are summarized in Supplementary Table S1.

#### 2.3.1 Baseline and linear models

This category covers foundational models that assume a linear relationship between predictors and outcomes. They serve as essential baselines for benchmarking complex algorithms and offer high interpretability for understanding data structure.

**Logistic regression** is a fundamental method rooted in biostatistics, widely used for analyzing binary outcomes like mortality (12). As a special case of Generalized Linear Models (GLMs), it models the log-odds of the outcome using a logit link: *z* = β_0_ + $\overset{p}{\underset{j=1}{\sum }}\beta_{j}X_{j}$, with *P*(*Y* = 1|*X*) = 1/(1 + *e*^−*z*^). The coefficients (β) are estimated by maximizing the log-likelihood. Exponentiated coefficients (*e*^β^) represent odds ratios, enabling intuitive interpretation. In this study, logistic regression serves as the baseline for evaluating more advanced models.

**Lasso**, short for Least Absolute Shrinkage and Selection Operator, enhances regression by adding an L1 penalty to the loss function: −[log-likelihood] + $\lambda \overset{p}{\underset{j=1}{\sum }}|\beta_{j}|$ (13). The penalty term encourages sparsity, shrinking some coefficients to exactly zero and enabling automatic feature selection. This is particularly useful for high-dimensional clinical data (14). The tuning parameter λ is selected via cross-validation, and efficient algorithms like coordinate descent enable scalable implementation (15).

**Elastic-net**, introduced by Zou and Hastie, addresses Lasso's limitations in handling correlated features and high-dimensional data (16). It blends L1 and L2 penalties in its cost function: $-\left[\text{log-likelihood}\right]+\lambda \left(\alpha \overset{p}{\underset{j=1}{\sum }}|\beta_{j}|+\frac{1-\alpha }{2}\overset{p}{\underset{j=1}{\sum }}\beta_{j}^{2}\right)$. The α parameter controls the balance between Lasso (α = 1) and Ridge (α = 0). Elastic-Net retains sparsity while encouraging grouped variable selection, ideal for domains with naturally clustered features (17). Coordinate descent efficiently computes regularization paths over λ and α.

**Linear discriminant analysis (LDA)**, developed by Fisher, is both a classifier and dimensionality reduction method. It assumes multivariate Gaussian predictors per class with shared covariance Σ, estimating posterior probabilities via Bayes' theorem. The decision rule is based on the discriminant score: $\delta_{k}\left(x\right)=x^{T}\sum^{-1}\mu_{k}-\frac{1}{2}\mu_{k}^{T}\sum^{-1}\mu_{k}$ + log(π_*k*_), where π_*k*_ is the class prior. Compared to logistic regression, LDA may perform better with small samples when its distributional assumptions hold (18). As a projection technique, LDA also maximizes between-class to within-class variance ratio for effective class separation (19).

#### 2.3.2 Tree-based ensemble models

This model family builds on decision trees by combining multiple trees into an ensemble to improve robustness and accuracy. Ensembling effectively captures complex non-linear patterns and reduces overfitting (20). We consider two main ensembling paradigms: bagging and boosting.

**Classification and regression tree (CART)**, introduced by Breiman et al., underpins most ensemble methods. It recursively partitions the feature space into high-dimensional rectangles via greedy, impurity-based splits (e.g., Gini impurity: $G=\overset{K}{\underset{k=1}{\sum }}{\hat{p}}_{mk}\left(1-{\hat{p}}_{mk}\right)$). Though interpretable, fully grown trees overfit and are sensitive to noise. *Post-hoc* cost-complexity pruning mitigates this (21). Despite instability, CART's flexibility and non-parametric nature make it a valuable standalone tool and a standard weak learner in ensemble models.

**Bagging-based ensembles** use Bootstrap Aggregating, proposed by Breiman to reduce model variance (22). Multiple base learners are trained on bootstrap samples and aggregated via majority voting. This stabilizes predictions while maintaining low bias.

- **Random forest**, a widely used bagging model, introduces dual randomness (23): each tree trains on a bootstrap sample, and each node split considers a random feature subset. This decorrelates trees and improves generalization. It also enables internal validation via out-of-bag (OOB) error estimation.
- **Extremely randomized trees (ExtraTrees)** further increases randomness (24). It selects split thresholds randomly and often uses the full dataset instead of bootstrapping. This yields faster training and lower variance, albeit sometimes with higher bias.

**Boosting-based ensembles** sequentially train weak learners to reduce bias. Each model corrects errors made by its predecessors, akin to functional gradient descent.

- **Adaptive boosting (AdaBoost)** fits learners on re-weighted data (25), increasing focus on previously misclassified instances. Final predictions are a weighted vote, favoring accurate learners. AdaBoost effectively minimizes exponential loss.
- **Gradient Boosting Decision Tree (GBDT)** generalizes boosting by directly optimizing a differentiable loss. Each tree fits the negative gradient (pseudo-residuals) of the loss. Stochastic subsampling and learning rate (shrinkage) improve generalization.
- **XGBoost** enhances GBDT with second-order optimization and regularization (26). It incorporates both gradient and Hessian terms, applies L1/L2 penalties, and introduces system-level optimizations (e.g., quantile sketch, sparsity-aware learning).
- **LightGBM** accelerates GBDT via Gradient-based One-Side Sampling (GOSS) and Exclusive Feature Bundling (EFB). It grows trees leaf-wise (best-first), focusing on splits that yield the most loss reduction.
- **CatBoost** specializes in handling categorical data. It uses permutation-based encoding to prevent target leakage, ordered boosting to control overfitting, and symmetric trees for efficient and regularized learning.

#### 2.3.3 Other classic non-linear models

This group includes widely used classifiers with distinct assumptions from linear or ensemble-based models, offering alternative paths for capturing non-linear patterns.

**Support Vector Machine (SVM)** is a discriminative classifier that finds the hyperplane maximizing the margin between classes, defined by support vectors (27). A soft margin introduces a regularization term *C*, balancing margin size and classification errors. The kernel trick enables non-linear separation by implicitly mapping inputs to higher-dimensional space using kernel functions such as linear, polynomial, or RBF: $K\left(x_{i},x_{j}\right)=\exp\left(-\gamma ∥x_{i}-x_{j}{∥}^{2}\right)$ (28). Hyperparameters *C* and γ control bias-variance trade-offs. Since SVM relies on distance calculations, input features must be scaled.

**K-Nearest Neighbors (KNN)** is a non-parametric, instance-based learner that classifies new samples based on the majority class among their *K* closest neighbors, using distance metrics like Euclidean or Hamming (29). Weighted KNN gives closer neighbors more influence. A smaller *K* increases variance, while a larger *K* smooths predictions. KNN is sensitive to feature scales and suffers in high-dimensional settings due to the "curse of dimensionality". Prediction is also computationally intensive, often mitigated via k-d trees or ball trees.

**Naive Bayes** is a generative classifier based on Bayes' theorem, making the naive assumption of conditional independence among features (30). This simplifies the likelihood to $\prod_{j=1}^{p}P\left(X_{j}|Y=k\right)$, making training efficient with limited data. Despite strong assumptions, it often performs well because only relative probabilities matter. Gaussian Naive Bayes assumes normally distributed features, while other variants like Multinomial NB apply Laplace smoothing for discrete features. Its success in medical diagnosis highlights its practical relevance.

#### 2.3.4 Neural network and deep learning models

This final category comprises the most complex models studied, capable of learning deep, abstract representations to uncover intricate non-linear patterns.

**Neural Network (NN)** here refers to a Multi-Layer Perceptron (MLP), consisting of an input layer, hidden layers with nonlinear activations (e.g., ReLU), and an output layer with sigmoid activation for binary classification. Training uses backpropagation with optimizers like Adam, minimizing binary cross-entropy loss. Dropout regularization randomly deactivates neurons during training to prevent overfitting. The architecture (layers, units, learning rate, dropout) is tuned for performance.

**Extreme Learning Machine (ELM)** is a single-hidden-layer feedforward network with randomly initialized, fixed hidden weights and analytically solved output weights (31). With sufficient hidden nodes and nonlinear activations, ELM retains universal approximation capability (32). Output weights are computed via Moore-Penrose pseudoinverse: β = *H*^†^*T*. Regularized ELM adds an L2 penalty for better generalization. Though fast, ELMs may require more hidden units and are sensitive to initializations (33).

**Long Short-Term Memory (LSTM)** is a gated recurrent neural network designed to capture long-range dependencies in sequences while mitigating vanishing gradients (34). Its memory cell uses three gates: forget, input, and output, each controlled by a sigmoid activation. These gates regulate what to retain, update, and output at each timestep. GRUs simplify LSTMs by combining gates. These gated architectures have become essential for sequential modeling in deep learning (35).

**Transformer** is a deep learning architecture that replaces recurrence with attention mechanisms to capture global dependencies. A standard Transformer consists of stacked encoder blocks, each with two sub-layers: multi-head self-attention and a position-wise feed-forward network. Residual connections and layer normalization follow each sub-layer to stabilize training. The core self-attention mechanism computes pairwise interactions via Query, Key, and Value projections; attention scores are derived from dot products between Queries and Keys. Multi-head attention enables parallel attention over multiple subspaces. Since the architecture lacks inherent order, positional encodings are added to input embeddings. Encoder-only models like BERT have proven highly effective in representation learning (36). Though originally for text, Transformers are increasingly adapted for tabular data through specialized embedding schemes for categorical and continuous features.

#### 2.3.5 Hyperparameter configuration

The optimal hyperparameters for the top-performing models were determined through 5-fold cross-validation on the training set. Table 3 presents the final hyperparameter configurations for the three best-performing gradient boosting models. These parameters were selected based on cross-validation performance and were subsequently used for final model evaluation on the held-out test set.

**Table 3:** Optimized hyperparameters for top-performing models.

**Model**	**Learning rate**	**Max depth**	**N estimators**	**Subsample**	**Num leaves**
XGBoost	0.05	3	100	0.8	-
AdaBoost	0.5	-	100	-	-
LightGBM	0.1	-	100	-	31

#### 2.3.6 Performance evaluation metrics

To comprehensively assess the predictive performance of the final models after hyperparameter optimization, we conducted evaluations on the held-out test set comprising 30% of the total data. Given the class imbalance characteristic inherent in clinical mortality prediction tasks, we adopted six widely accepted metrics for binary classification, each offering distinct insights based on the confusion matrix components: True Positives (TP), True Negatives (TN), False Positives (FP), and False Negatives (FN).

**Accuracy** quantifies the overall proportion of correctly classified samples:


$$\text{Accuracy}=\frac{TP+TN}{TP+TN+FP+FN}.$$

Although intuitive, accuracy can be misleading in imbalanced scenarios, where high values may arise from dominant majority-class predictions, masking poor minority-class performance.

**Precision (positive predictive value)** measures the proportion of true positives among all predicted positives:


$$\text{Precision}=\frac{TP}{TP+FP}.$$

In clinical contexts, high precision ensures that most individuals predicted as high-risk (i.e., mortality-positive) are indeed true cases, thereby minimizing false alarms and resource misallocation.

**Recall (sensitivity, true positive rate)** evaluates the model's ability to capture all actual positive cases:


$$\text{Recall}=\frac{TP}{TP+FN}.$$

It is critical in medical applications where failing to identify high-risk individuals (false negatives) could result in severe clinical consequences.

**F1 score** defined as the harmonic mean of precision and recall, provides a single aggregated metric balancing both types of error:


$$\text{F1 Score}=2\times \frac{\text{Precision}\times \text{Recall}}{\text{Precision}+\text{Recall}}=\frac{2TP}{2TP+FP+FN}.$$

It is particularly informative for imbalanced datasets where disproportionate performance on either metric must be penalized.

**Area under the ROC curve (ROC AUC)** The ROC AUC measures the model's discriminative capability across all classification thresholds. The ROC curve plots the True Positive Rate (TPR) against the False Positive Rate (FPR):


$$\text{FPR}=\frac{FP}{FP+TN}.$$

A higher AUC indicates better overall ranking performance, reflecting the probability that a randomly selected positive instance is ranked higher than a randomly selected negative instance.

**Area under the precision recall curve (PR AUC)** focuses on the trade-off between precision and recall across varying thresholds. Unlike ROC AUC, which may be overly optimistic under class imbalance, PR AUC provides a more realistic estimate of the model's ability to identify true positives within the minority class:


$$\text{PR AUC}=\int_{0}^{1}\text{Precision}\left(\text{Recall}\right)d\left(\text{Recall}\right).$$

This metric is particularly valuable for clinical mortality prediction, where the positive class (e.g., adverse outcomes) is typically underrepresented.

All experiments were conducted in Python (v3.11) within isolated Conda environments. The primary libraries and frameworks is available in Supplementary Figure S2.

## 3 Results

### 3.1 Baseline performance

Among the cohort, 123 patients (22.9%) died during follow-up (Non-survivors). Compared with survivors (*n* = 415), non-survivors were significantly older and had a higher proportion of males. Comorbidities such as diabetes and cardiovascular disease were more prevalent in non-survivors. Laboratory parameters revealed that non-survivors exhibited a state of heightened inflammation, evidenced by significantly higher C-reactive protein levels, and poorer nutritional status, indicated by lower levels of albumin, hemoglobin, and creatinine. Furthermore, non-survivors had a shorter dialysis vintage, higher body weight, lower pre-dialysis diastolic blood pressure, and lower triglyceride levels (Supplementary Table S3).

### 3.2 Overall performance distribution

Tables 4–6 present the performance results of 19 machine learning models across three evaluation metrics: Accuracy, F1 Score, and ROC AUC. In general, tree-based ensemble models–particularly those based on gradient boosting—tended to achieve higher scores across the majority of experiments. Models such as XGBoost, AdaBoost, CatBoost, and LightGBM frequently ranked among the top-performing approaches, while linear models, traditional classifiers, and neural network-based models exhibited more variable performance in this clinical prediction context.

**Table 4:** Performance comparison of 19 machine learning models (optimized for accuracy).

**Model**	**ROC**	**PR**	**F1 score**	**Accuracy**	**Precision**	**Recall**
XGBoost	**0.899**	0.677	**0.683**	**0.852**	0.667	0.70
CatBoost	0.899	0.684	0.606	0.852	0.769	0.50
Gradient Boosting Decision Tree	0.896	0.686	0.581	0.852	**0.818**	0.45
LightGBM	0.886	**0.762**	0.667	0.852	0.684	0.65
Random Forest	0.874	0.685	0.651	0.830	0.609	0.70
ExtraTrees	0.864	0.684	0.562	0.841	0.750	0.45
AdaBoost	0.840	0.639	0.516	0.830	0.727	0.40
KNN	0.819	0.646	0.545	0.830	0.692	0.45
LSTM	0.810	0.667	0.556	0.818	0.625	0.50
SVM	0.804	0.599	0.541	0.807	0.588	0.50
Linear Discriminant Analysis	0.803	0.573	0.556	0.818	0.625	0.50
Elastic-Net	0.799	0.553	0.571	0.830	0.667	0.50
Lasso	0.796	0.553	0.571	0.830	0.667	0.50
Logistic Regression	0.796	0.553	0.571	0.830	0.667	0.50
Extreme Learning Machine	0.788	0.542	0.606	0.852	0.769	0.50
Neural Network	0.784	0.568	0.556	0.818	0.625	0.50
Naive Bayes	0.771	0.536	0.391	0.364	0.250	**0.90**
Decision Tree (CART)	0.734	0.473	0.541	0.807	0.588	0.50
Transformer	0.655	0.463	0.455	0.727	0.417	0.50

The highest value in each metric column is highlighted in bold.

**Table 5:** Performance comparison of 19 machine learning models (optimized for F1 score).

**Model**	**ROC**	**PR**	**F1 score**	**Accuracy**	**Precision**	**Recall**
AdaBoost	**0.903**	0.709	**0.682**	**0.841**	0.625	0.75
LightGBM	0.886	**0.758**	0.636	0.818	0.583	0.70
CatBoost	0.882	0.685	0.632	0.841	0.667	0.60
Random Forest	0.847	0.703	0.627	0.784	0.516	0.80
Gradient Boosting Decision Tree	0.873	0.662	0.588	0.841	0.714	0.50
ExtraTrees	0.864	0.684	0.562	0.841	**0.750**	0.45
SVM	0.768	0.627	0.558	0.784	0.522	0.60
XGBoost	0.854	0.610	0.550	0.795	0.550	0.55
Extreme Learning Machine	0.799	0.540	0.545	0.830	0.692	0.45
KNN	0.819	0.646	0.545	0.830	0.692	0.45
Elastic-Net	0.799	0.516	0.545	0.716	0.429	0.75
Lasso	0.795	0.499	0.536	0.705	0.417	0.75
Logistic Regression	0.800	0.506	0.536	0.705	0.417	0.75
Decision Tree (CART)	0.683	0.405	0.533	0.761	0.480	0.60
Linear Discriminant Analysis	0.782	0.555	0.512	0.761	0.478	0.55
LSTM	0.805	0.691	0.500	0.773	0.500	0.50
Transformer	0.746	0.449	0.486	0.784	0.529	0.45
Naive Bayes	0.771	0.536	0.391	0.364	0.250	**0.90**
Neural Network	0.737	0.508	0.000	0.761	0.000	0.00

The highest value in each metric column is highlighted in bold.

**Table 6:** Performance Comparison of 19 Machine Learning Models (Optimized for ROC AUC).

**Model**	**ROC**	**PR**	**F1 score**	**Accuracy**	**Precision**	**Recall**
CatBoost	**0.898**	0.687	0.545	0.830	0.692	0.450
AdaBoost	0.894	0.695	**0.684**	**0.864**	0.722	0.650
LightGBM	0.882	**0.750**	0.651	0.830	0.609	0.700
XGBoost	0.868	0.670	0.579	0.818	0.611	0.550
Random Forest	0.868	0.682	0.596	0.784	0.519	0.700
ExtraTrees	0.857	0.668	0.500	0.818	0.667	0.400
Gradient Boosting Decision Tree	0.836	0.643	0.516	0.830	0.727	0.400
SVM	0.827	0.560	0.578	0.784	0.520	0.650
Neural Network	0.813	0.624	0.500	0.818	0.667	0.400
Linear Discriminant Analysis	0.812	0.589	0.541	0.807	0.588	0.500
Lasso	0.802	0.537	0.545	0.830	0.692	0.450
Logistic Regression	0.802	0.537	0.545	0.830	0.692	0.450
Elastic-Net	0.801	0.558	0.606	0.852	0.769	0.500
LSTM	0.801	0.666	0.579	0.818	0.611	0.550
Transformer	0.788	0.625	0.529	0.818	0.643	0.450
Extreme Learning Machine	0.772	0.527	0.588	0.841	0.714	0.500
Naive Bayes	0.771	0.536	0.391	0.364	0.250	**0.900**
Decision Tree(CART)	0.764	0.460	0.320	0.807	**0.800**	0.200
KNN	0.760	0.567	0.516	0.830	0.727	0.400

The highest value in each metric column is highlighted in bold.

**Figure 1:**
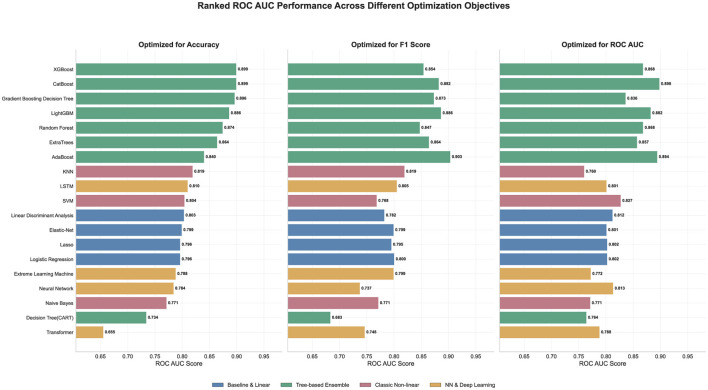
Comparison of machine learning model performance across different optimization metrics. The horizontal bar chart displays ROC AUC scores for various algorithms optimized for accuracy **(left)**, F1 score **(middle)**, and ROC AUC **(right)**. Models are categorized into four groups: Baseline & Linear (blue), Tree-based Ensemble (green), Classic Non-linear (pink), and Neural Network & Deep Learning (orange).

The comprehensive evaluation across different optimization strategies reveals significant performance variations, as illustrated in Figure 1. Tree-based ensemble methods demonstrate superior performance across all three optimization objectives, with XGBoost, CatBoost, and Gradient Boosting Decision Tree consistently achieving ROC AUC scores above 0.89 when optimized for accuracy. Notably, XGBoost maintains exceptional consistency, delivering ROC AUC scores of 0.899, 0.894, and 0.868 when optimized for accuracy, F1 score, and ROC AUC respectively. The ensemble methods collectively outperform other algorithm categories, with CatBoost achieving the highest ROC AUC score of 0.898 when optimized for ROC AUC itself. Traditional machine learning algorithms show moderate performance, with Random Forest achieving competitive results (ROC AUC of 0.874 for accuracy optimization), while baseline linear methods such as Logistic Regression, Lasso, and Elastic-Net cluster around 0.79–0.80 ROC AUC scores regardless of optimization strategy. Deep learning approaches exhibit mixed results, with Neural Networks showing better performance (ROC AUC around 0.78–0.81) compared to the more complex Transformer architecture, which achieved only 0.655–0.748 across different optimization metrics.

**Figure 2:**
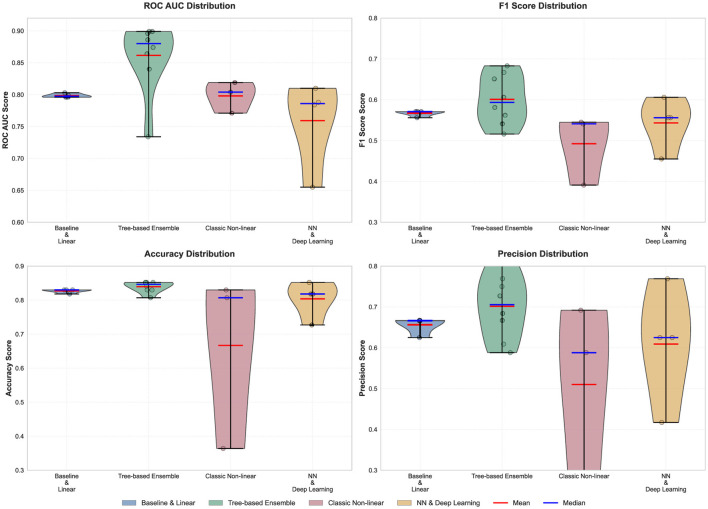
Distribution of performance metrics across algorithm categories using violin plots. The figure shows the probability density distribution for ROC AUC (**top left**), F1 Score (**top right**), Accuracy (**bottom left**), and Precision (**bottom right**). Each violin plot displays the full distribution shape with median (blue line) and mean (red line) indicators for four algorithm categories: Baseline & Linear, Tree-based Ensemble, Classic Non-linear, and Neural Network & Deep Learning.

The distribution analysis presented in Figure 2 provides deeper insights into the variability and consistency of different algorithm categories across multiple performance metrics. Tree-based ensemble methods exhibit the most favorable performance characteristics, demonstrating both high median performance and relatively tight distributions across all metrics, with ROC AUC values concentrated around 0.87–0.89 and F1 scores clustering near 0.60. The violin plots reveal that tree-based ensembles maintain consistent performance with minimal variance, indicating their reliability across different evaluation criteria.

In contrast, classic non-linear methods display the highest variability, particularly evident in the accuracy and precision distributions, where the violin shapes show broad, bimodal characteristics spanning from approximately 0.35–0.85 for accuracy. This high variance suggests that classic non-linear algorithms like SVM, KNN, and Naive Bayes are highly sensitive to hyperparameter settings and data characteristics. Baseline and linear methods demonstrate consistent but modest performance with narrow distributions, showing predictable behavior with ROC AUC values tightly clustered around 0.80 and F1 scores near 0.57. Neural network and deep learning approaches exhibit moderate performance with considerable spread, particularly in precision metrics where the distribution ranges from 0.42 to 0.77, indicating that while these methods can achieve competitive results, they require careful tuning and may be more sensitive to training conditions and architecture choices.

To assess the statistical robustness of the top-performing models, we computed 95% confidence intervals for ROC AUC using bootstrap resampling of the test set (1,000 iterations). Table 7 presents the bootstrap confidence intervals for the three best-performing gradient boosting models. All three models demonstrated robust and statistically reliable performance, with narrow confidence intervals indicating stable discrimination capability across resampled test sets.

**Table 7:** Bootstrap confidence intervals (95%) for top-performing models based on 1,000 bootstrap iterations.

**Model**	**Mean AUC**	**95% CI lower**	**95% CI upper**
XGBoost	0.850	0.795	0.904
AdaBoost	0.845	0.790	0.901
LightGBM	0.840	0.785	0.897

The comprehensive evaluation across different optimization strategies reveals significant performance variations, as illustrated in Figure 1. Tree-based ensemble methods demonstrate superior performance across all three optimization objectives, with XGBoost, CatBoost, and Gradient Boosting Decision Tree consistently achieving ROC AUC scores above 0.89 when optimized for accuracy. Notably, XGBoost maintains exceptional consistency, delivering ROC AUC scores of 0.899, 0.894, and 0.868 when optimized for accuracy, F1 score, and ROC AUC respectively. The ensemble methods collectively outperform other algorithm categories, with CatBoost achieving the highest ROC AUC score of 0.898 when optimized for ROC AUC itself. Traditional machine learning algorithms show moderate performance, with Random Forest achieving competitive results (ROC AUC of 0.874 for accuracy optimization), while baseline linear methods such as Logistic Regression, Lasso, and Elastic-Net cluster around 0.79–0.80 ROC AUC scores regardless of optimization strategy. Deep learning approaches exhibit mixed results, with Neural Networks showing better performance (ROC AUC around 0.78–0.81) compared to the more complex Transformer architecture, which achieved only 0.655–0.748 across different optimization metrics.

The distribution analysis presented in Figure 2 provides deeper insights into the variability and consistency of different algorithm categories across multiple performance metrics. Tree-based ensemble methods exhibit the most favorable performance characteristics, demonstrating both high median performance and relatively tight distributions across all metrics, with ROC AUC values concentrated around 0.87–0.89 and F1 scores clustering near 0.60. The violin plots reveal that tree-based ensembles maintain consistent performance with minimal variance, indicating their reliability across different evaluation criteria.

In contrast, classic non-linear methods display the highest variability, particularly evident in the accuracy and precision distributions, where the violin shapes show broad, bimodal characteristics spanning from approximately 0.35–0.85 for accuracy. This high variance suggests that classic non-linear algorithms like SVM, KNN, and Naive Bayes are highly sensitive to hyperparameter settings and data characteristics. Baseline and linear methods demonstrate consistent but modest performance with narrow distributions, showing predictable behavior with ROC AUC values tightly clustered around 0.80 and F1 scores near 0.57. Neural network and deep learning approaches exhibit moderate performance with considerable spread, particularly in precision metrics where the distribution ranges from 0.42 to 0.77, indicating that while these methods can achieve competitive results, they require careful tuning and may be more sensitive to training conditions and architecture choices.

### 3.3 Optimization objective impact analysis

#### 3.3.1 Accuracy optimized models

When models were tuned to maximize accuracy, the **XGBoost** model demonstrated the best overall performance, achieving a leading ROC AUC of 0.899 and the highest F1 Score of 0.683, coupled with a top-tier accuracy of 0.852. Other boosting models also performed exceptionally well, with Gradient Boosting Decision Tree (GBDT) recording the highest precision (0.818), although this came at the expense of a lower recall (0.45), highlighting a classic precision-recall trade-off. In contrast, the Naive Bayes model yielded an extremely high recall (0.90) but at the cost of very poor precision (0.250) and accuracy (0.364), suggesting a tendency to frequently predict the positive class.

#### 3.3.2 F1-score optimized models

When targeting the F1 Score, the **AdaBoost** model emerged as the definitive top performer, achieving the highest F1 Score (0.682) and accuracy (0.841), along with an outstanding ROC AUC of 0.903. This result underscores that the choice of metric during hyperparameter tuning can substantially alter a model's final behavior and ranking. LightGBM also demonstrated exceptional performance, registering the highest PR AUC (0.758), confirming its robustness in handling the minority (mortality) class. Notably, under this optimization scheme, the standard Neural Network model failed to produce a useful classifier, resulting in an F1 Score of 0.0, suggesting the model converged on a trivial solution of predicting only the majority class.

#### 3.3.3 ROC AUC optimized models

In experiments designed to maximize the ROC AUC, a nuanced picture of model performance emerged. While **CatBoost** technically achieved the highest ROC AUC (0.898), the **AdaBoost** model presented a more compelling and clinically practical performance group. Despite a marginally lower ROC AUC (0.894), AdaBoost delivered a substantially better F1 Score (0.684 vs. 0.545 for CatBoost) and higher accuracy (0.864 vs. 0.830). This finding suggests that the model with the highest score on a single threshold-independent metric like ROC AUC may not be the most balanced or useful model when evaluated on threshold-dependent metrics like F1 Score, which are often more relevant for clinical decision-making.

### 3.4 Systematic clinical feature group ablation analysis

To investigate the contribution of different clinical domains to mortality prediction in maintenance hemodialysis patients, we conducted a comprehensive ablation study. This analysis systematically evaluates how the removal of specific clinical feature groups affects model performance across multiple machine learning architectures.

The ablation study encompassed 570 individual experiments, evaluating 10 distinct clinical feature groups across 19 machine learning algorithms under three optimization objectives (10 × 19 × 3 = 570). This approach enables quantitative assessment of each clinical domain's predictive value and identification of potential feature interdependencies.

#### 3.4.1 Ablation design

To systematically assess the contribution of clinical feature clusters, variables were categorized into 10 domains based on physiological functions and clinical relevance in hemodialysis patients (Table 8): baseline demographics, comorbidity profile, vascular/hemodynamic status, inflammatory-immune markers, dialysis adequacy/residual renal function, metabolic homeostasis, mineral-bone disorder, nutritional status, hematological function/anemia, and hemodialysis key indicators. This classification enables targeted ablation analysis.

**Table 8:** Clinical feature groups for systematic ablation analysis.

**Ablation ID**	**Clinical domain**	**Key features removed**	**Clinical rationale**
*Abl*_1_	*General information*	Age, Sex, Weight	Baseline demographic
*Abl*_2_	*Comorbidity profile*	Hypertension, Diabetes, Cardiovascular diseases	Common complications
*Abl*_3_	*Blood pressure*	Hypertension, Systolic pressure before dialysis, Diastolic blood pressure before dialysis	Vascular status and hemodynamics
*Abl*_4_	*Inflammation markers*	Neutrophils, C-reactive protein, Lymphocyte, Albumin, β-Microglobulin	Inflammatory immune markers
*Abl*_5_	*Dialysis efficiency*	24-h urine output, Creatinine, Urea nitrogen, SpKT/V	Dialysis adequacy and residual renal function
*Abl*_6_	*Internal environment*	Sodium, Potassium, Calcium, Phosphorus, Carbon dioxide	Metabolic homeostasis and uremic toxin burden
*Abl*_7_	*Bone metabolism*	Phosphorus, Calcium, Parathyroid hormone	Mineral bone disorder indicators
*Abl*_8_	*Nutritional status*	Uric acid, Albumin, Cholesterol, Triglyceride, Blood sugar	Malnutrition and protein-energy wasting markers
*Abl*_9_	*Blood cell profiles*	Neutrophils, Lymphocyte, Platelet, Hemoglobin, Ferritin	Hematological status and anemia assessment
*Abl*_10_	*Hemodialysis indicators*	First vascular access, β-Microglobulin, Dialysis period, SpKT/V	Duration, access, and adequacy of hemodialysis

#### 3.4.2 Performance analysis

Tables 9–11 summarize the performance of 19 models under the original and ten ablation settings, evaluated by Precision-Recall (PR), F1 Score, and ROC AUC, respectively. Overall, tree-based ensemble models—such as LightGBM, XGBoost, and CatBoost—tended to achieve higher and more stable scores across different metrics and ablations. In contrast, the performance of neural networks and linear models exhibited greater variability depending on the ablated feature sets. These results suggest a relative robustness of ensemble methods in this clinical prediction task, particularly under conditions of partial feature removal (Supplementary Table S1).

**Table 9:** Precision-Recall scores for each model under the original and ten ablation settings.

**Model**	**Base**	***Abl*_1_**	***Abl*_2_**	***Abl*_3_**	***Abl*_4_**	***Abl*_5_**	***Abl*_6_**	***Abl*_7_**	***Abl*_8_**	***Abl*_9_**	***Abl*_10_**
XGBoost	0.671	0.707	0.606	0.649	0.656	0.660	0.654	0.625	0.557	**0.737**	0.640
Transformer	0.463	0.544	0.502	0.658	0.590	0.518	0.517	0.505	0.537	0.430	0.546
SVM	0.600	0.522	0.570	0.527	0.567	0.570	0.567	0.604	0.590	0.554	0.619
Random Forest	0.685	0.640	0.640	0.607	0.644	0.681	0.698	0.688	0.643	0.683	0.665
Neural Network	0.568	0.495	0.581	0.533	0.543	0.638	0.537	0.529	0.580	0.548	**0.714**
Naive Bayes	0.537	0.487	0.511	0.487	0.492	0.646	0.547	0.528	0.508	0.511	0.621
LSTM	0.667	0.637	0.597	0.647	0.506	0.605	0.658	0.708	0.567	0.538	0.557
Logistic Regression	0.554	0.512	0.547	0.520	0.517	0.571	0.543	0.539	0.534	0.551	0.621
Linear Discriminant	0.574	0.520	0.545	0.559	0.553	0.587	0.586	0.571	0.569	0.557	0.608
LightGBM	**0.762**	**0.721**	**0.709**	0.702	**0.707**	**0.737**	**0.757**	**0.735**	0.643	0.730	0.636
Lasso	0.554	0.517	0.547	0.526	0.518	0.571	0.542	0.539	0.534	0.547	0.599
KNN	0.646	0.541	0.553	0.578	0.517	0.613	0.578	0.562	0.614	0.570	0.594
Gradient	0.686	0.645	0.666	0.645	0.660	0.681	0.690	0.639	**0.662**	0.676	0.657
Extreme Learning	0.542	0.530	0.575	0.564	0.513	0.571	0.534	0.563	0.536	0.572	0.604
Extra Trees	0.684	0.626	0.680	**0.720**	0.636	0.668	0.673	0.671	0.639	0.667	0.663
Elastic-net	0.553	0.541	0.550	0.551	0.518	0.572	0.554	0.544	0.538	0.539	0.603
Decision Tree	0.473	0.532	0.472	0.412	0.643	0.421	0.497	0.372	0.464	0.494	0.490
CatBoost	0.684	0.639	0.680	0.630	0.647	0.707	0.641	0.658	0.639	0.704	0.530
Adaboost	0.639	0.574	0.639	0.661	0.608	0.602	0.741	0.603	0.546	0.634	0.697

The highest value for each model is highlighted in bold.

**Table 10:** F1 scores for each model under the original and ten ablation settings.

**Model**	**Base**	***Abl*_1_**	***Abl*_2_**	***Abl*_3_**	***Abl*_4_**	***Abl*_5_**	***Abl*_6_**	***Abl*_7_**	***Abl*_8_**	***Abl*_9_**	***Abl*_10_**
XGBoost	0.550	0.634	0.541	0.541	0.588	0.579	0.537	0.526	0.650	0.632	0.513
Transformer	0.486	0.458	0.424	0.571	0.500	0.485	0.564	0.579	0.486	0.529	0.457
SVM	0.558	0.552	0.526	0.605	0.558	0.558	0.526	0.500	0.524	0.605	0.538
Random Forest	0.627	0.571	0.622	0.609	0.545	0.683	0.531	0.605	0.634	0.609	0.627
Neural Network	0.500	0.564	0.500	0.452	0.545	0.541	0.485	0.500	0.545	0.541	0.308
Naive Bayes	0.391	0.391	0.391	0.383	0.387	0.400	0.418	0.413	0.371	0.391	0.387
LSTM	0.500	0.537	0.636	0.550	0.526	0.465	0.526	0.476	0.579	0.524	0.429
Logistic Regression	0.536	0.536	0.545	0.560	0.490	0.536	0.545	0.529	0.471	0.471	0.593
Linear Discriminant Analysis	0.512	0.481	0.560	0.542	0.490	0.524	0.524	0.524	0.512	0.524	0.529
LightGBM	0.636	0.667	0.650	0.650	**0.606**	0.683	0.591	0.634	**0.651**	0.683	0.619
Lasso	0.536	0.536	0.536	0.536	0.471	0.536	0.545	0.566	0.471	0.471	0.566
KNN	0.545	0.429	0.545	0.500	0.500	0.514	0.545	0.471	0.545	0.545	0.500
Gradient	0.588	**0.670**	0.595	0.541	0.529	0.588	**0.629**	0.649	0.514	0.650	0.556
Extreme Learning	0.545	0.571	0.471	0.588	0.562	0.471	0.485	0.529	0.438	0.588	0.444
Extra Trees	0.562	0.514	0.516	0.606	0.581	0.485	0.529	0.529	0.545	0.588	0.412
Elastic-net	0.545	0.536	0.536	0.545	0.471	0.536	0.545	0.538	0.542	0.526	0.604
Decision Tree	0.533	0.591	0.536	0.526	0.571	0.615	0.400	0.500	0.571	0.548	0.419
CatBoost	0.632	0.514	0.529	0.500	0.452	0.650	0.541	0.579	0.600	0.526	0.387
Adaboost	**0.682**	0.541	**0.682**	**0.718**	0.514	**0.727**	0.619	**0.714**	0.400	**0.684**	**0.682**

The highest value for each model is highlighted in bold.

**Table 11:** ROC AUC Results for each model under the original and ten ablation settings.

**Model**	**Base**	***Abl*_1_**	***Abl*_2_**	***Abl*_3_**	***Abl*_4_**	***Abl*_5_**	***Abl*_6_**	***Abl*_7_**	***Abl*_8_**	***Abl*_9_**	***Abl*_10_**
XGBoost	0.868	0.850	0.872	0.880	0.810	0.856	0.870	0.865	**0.858**	0.886	0.829
Transformer	0.787	0.757	0.835	0.707	0.776	0.806	0.767	0.816	0.726	0.747	0.698
SVM	0.827	0.798	0.826	0.823	0.774	0.738	0.788	0.828	0.800	0.816	0.782
Random Forest	0.868	0.878	0.830	0.873	0.793	0.868	0.874	0.879	0.835	0.856	0.846
Neural Network	0.813	0.761	0.786	0.799	0.750	0.817	0.748	0.801	0.812	0.816	0.854
Naive Bayes	0.771	0.761	0.753	0.764	0.743	0.800	0.804	0.766	0.755	0.763	0.772
LSTM	0.801	0.848	0.848	0.810	0.688	0.792	0.777	0.806	0.764	0.774	0.780
Logistic Regression	0.802	0.780	0.790	0.805	0.761	0.813	0.801	0.798	0.782	0.799	0.825
Linear Discriminant Analysis	0.812	0.792	0.793	0.811	0.771	0.793	0.815	0.806	0.796	0.795	0.815
LightGBM	0.882	0.880	0.883	0.884	**0.839**	0.886	**0.894**	0.887	0.857	**0.900**	0.863
Lasso	0.802	0.780	0.790	0.815	0.765	0.801	0.803	0.798	0.780	0.810	0.827
KNN	0.760	0.837	0.782	0.735	0.717	0.796	0.767	0.771	0.717	0.770	0.774
Gradient	0.836	0.837	0.837	0.835	0.829	0.847	0.839	0.839	0.824	0.846	0.874
Extreme Learning	0.772	0.806	0.850	0.817	0.758	0.783	0.804	0.853	0.783	0.730	0.812
Extra Trees	0.857	0.840	0.838	0.883	0.810	0.877	0.843	0.835	0.807	0.841	0.844
Elastic-net	0.801	0.782	0.792	0.807	0.765	0.807	0.801	0.802	0.788	0.810	0.825
Decision Tree	0.764	0.764	0.764	0.803	0.731	0.782	0.764	0.764	0.778	0.797	0.764
CatBoost	**0.898**	**0.884**	0.879	**0.887**	0.796	**0.912**	0.571	**0.899**	0.856	0.891	0.851
Adaboost	0.894	0.860	**0.894**	0.880	0.823	0.894	0.881	0.831	0.841	0.886	**0.893**

The highest value for each model is highlighted in bold.

**Figure 3:**
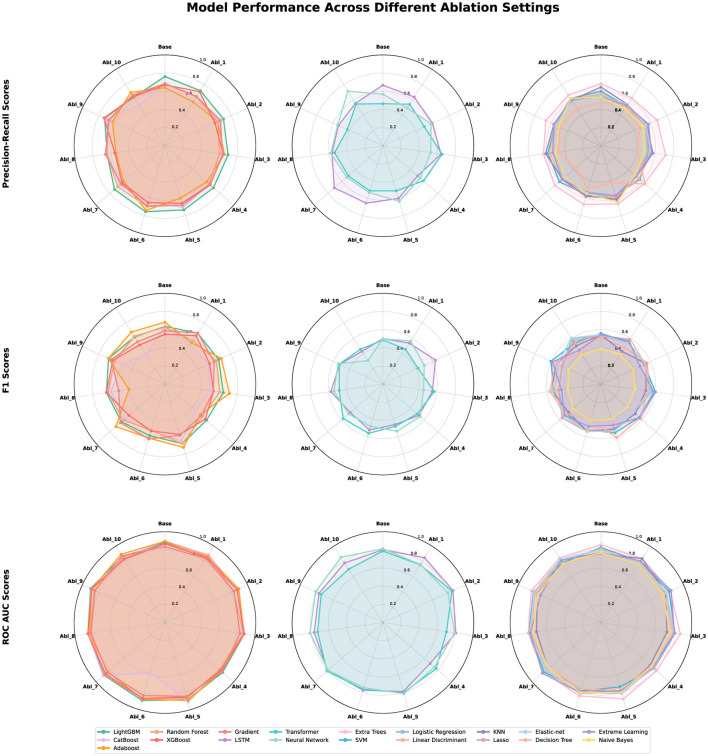
Combined radar charts illustrating model performance across systematic ablation settings for three evaluation metrics: Precision-Recall (**top**), F1 Score (**middle**), and ROC AUC (**bottom**). Each column represents a model category: ensemble-based methods, sequential or deep models, and linear or shallow classifiers. Polygons correspond to individual models, with vertices indicating baseline and ten ablation conditions. Larger radii reflect better performance.

Figure 3 summarizes the comparative robustness of machine learning models under progressive feature ablation. Tree-based ensembles such as LightGBM, CatBoost, Random Forest, and XGBoost maintain nearly circular patterns across all metrics, suggesting low dependence on specific feature groups. Sequential models including LSTM and Transformer display moderate deformations, indicating partial reliance on certain feature domains. Linear models such as Logistic Regression, Lasso, and Linear Discriminant Analysis show the most pronounced contractions, reflecting reduced stability when input features are removed. Overall, ensemble learners demonstrate the strongest tolerance to missing clinical information, whereas linear and sequence-based approaches are more sensitive to incomplete representations.

#### 3.4.3 Clinical domain-specific impacts

The ablation results reveal that the predictive importance of clinical domains varies considerably across feature groups. Among them, inflammation markers (Ablation 4) consistently led to performance degradation across nearly all models, with average ROC AUC reductions such as 0.058 for XGBoost and 0.043 for LightGBM, underscoring the central prognostic role of chronic inflammation in hemodialysis mortality. In contrast, the removal of nutritional status features (Ablation 8) resulted in more model-specific responses: XGBoost showed a drop in precision-recall performance, while Random Forest remained largely unaffected, suggesting potential overfitting or redundancy in some algorithms. Notably, eliminating blood cell profile features (Ablation 9) unexpectedly improved model performance in some cases—for example, XGBoost achieved its highest PR score (0.737) and LightGBM showed an AUC gain (0.882 → 0.900)—implying that these hematological features may introduce noise or multicollinearity. Hemodialysis indicators (Ablation 10), such as vascular access and dialysis duration, led to modest performance gains in neural networks but had little effect on ensemble models, likely due to their ability to capture compensatory patterns. In contrast, domains like blood pressure (Ablation 3) and bone metabolism (Ablation 7) had minimal impact across all metrics and algorithms, indicating lower standalone predictive value. These findings suggest that while some clinical groups—particularly inflammation and comorbidity—are indispensable, others may be partially redundant or less discriminative when evaluated in multivariate settings.

### 3.5 Model and result visualization and analysis

We used XGBoost machine learning to analyze dialysis patient data and created visualizations to understand which factors are most important for patient outcomes. We used SHAP (a method to explain AI decisions) to make the model's predictions understandable.

#### 3.5.1 Which factors matter most?

**Figure 4:**
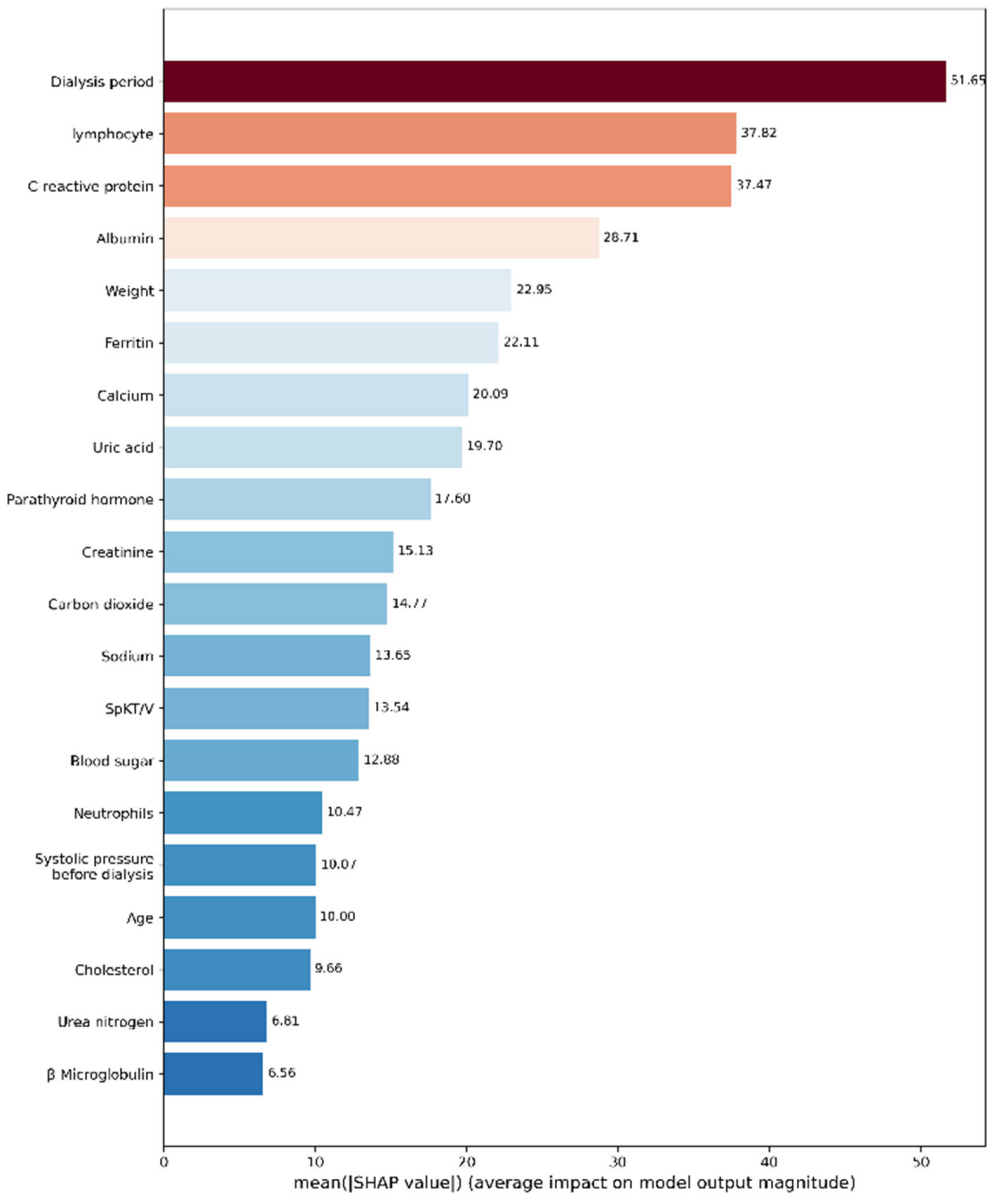
Top 20 most important factors affecting patient outcomes. Higher values mean the factor has bigger impact on predictions.

Figure 4 shows which patient factors are most important for predictions. The top three are:

- **Dialysis period** (51.65): How long the patient has been on dialysis is the most important factor
- **Lymphocyte count** (37.82): White blood cells that fight infection
- **C-reactive protein** (37.47): A marker that shows inflammation in the body

This tells us that time on dialysis and the patient's immune system health are crucial for outcomes.

#### 3.5.2 How do these factors affect outcomes?

**Figure 5:**
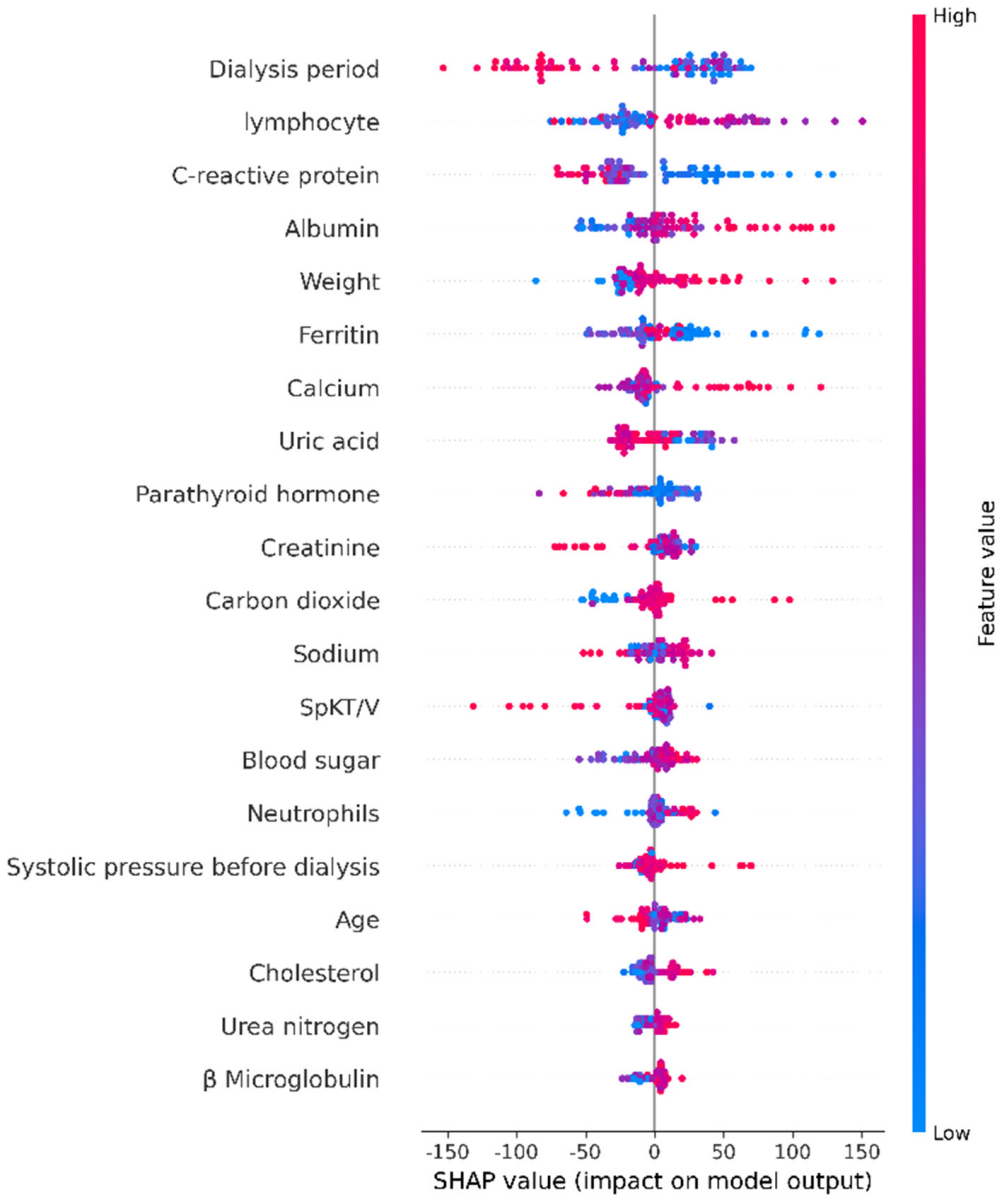
How each factor affects predictions. Red dots = high values, blue dots = low values. Points to the right improve outcomes, points to the left worsen outcomes.

Figure 5 shows a more detailed view:

- **Dialysis period**: Patients on dialysis longer (red dots) have worse outcomes (points are on the left)
- **Lymphocyte count**: The relationship is complex - both very high and very low counts can be problematic
- **C-reactive protein**: High inflammation (red dots) clearly leads to worse outcomes

#### 3.5.3 Understanding individual patient predictions

**Figure 6:**
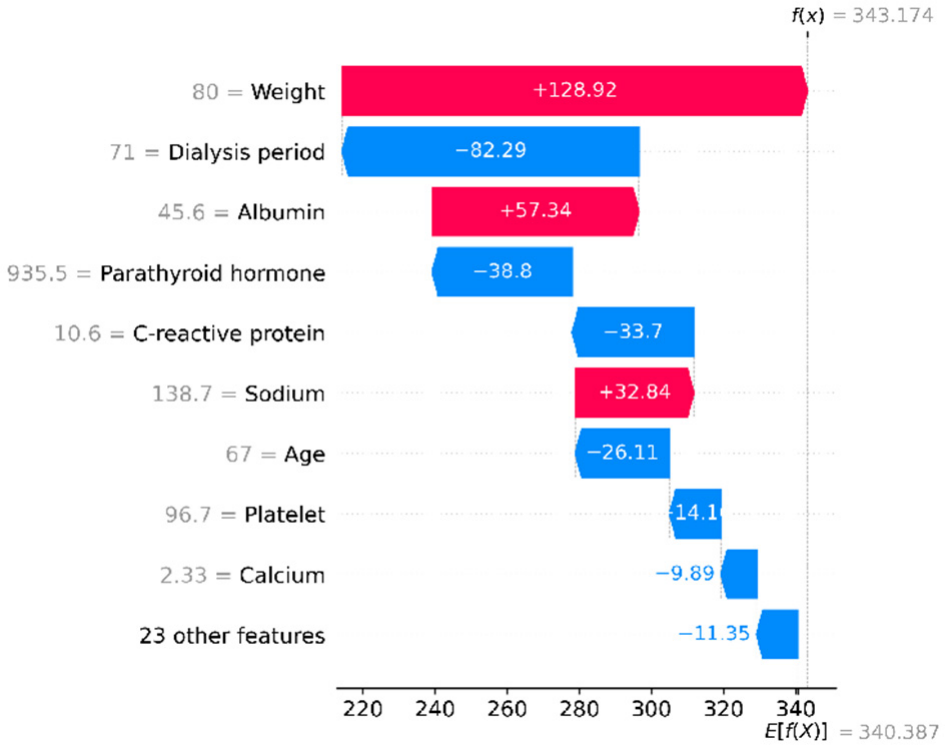
Example showing how different factors add up to predict one patient's outcome. Starting from baseline (340.387) to final prediction (343.174).

**Figure 7:**
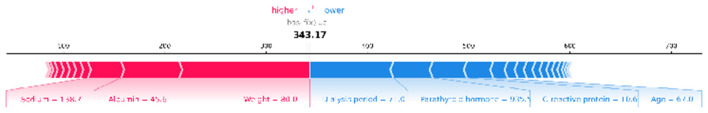
Another view of the same patient—red factors improve the prediction, blue factors worsen it.

Figures 6 and 7 show how we predict outcomes for one specific patient:

- **Good factors** (improving prediction): - Weight of 80 kg (+128.92)—healthy weight is beneficial - Albumin of 45.6 (+57.34)–good nutrition status - Sodium of 138.7 (+32.84)—normal electrolyte balance
- **Bad factors** (worsening prediction): - 71 months on dialysis (−82.29)—long time on dialysis - High parathyroid hormone of 935.5 (−38.8)—bone/mineral problems - C-reactive protein of 10.6 (−33.7)—inflammation present

#### 3.5.4 How factors change with different values

**Figure 8:**
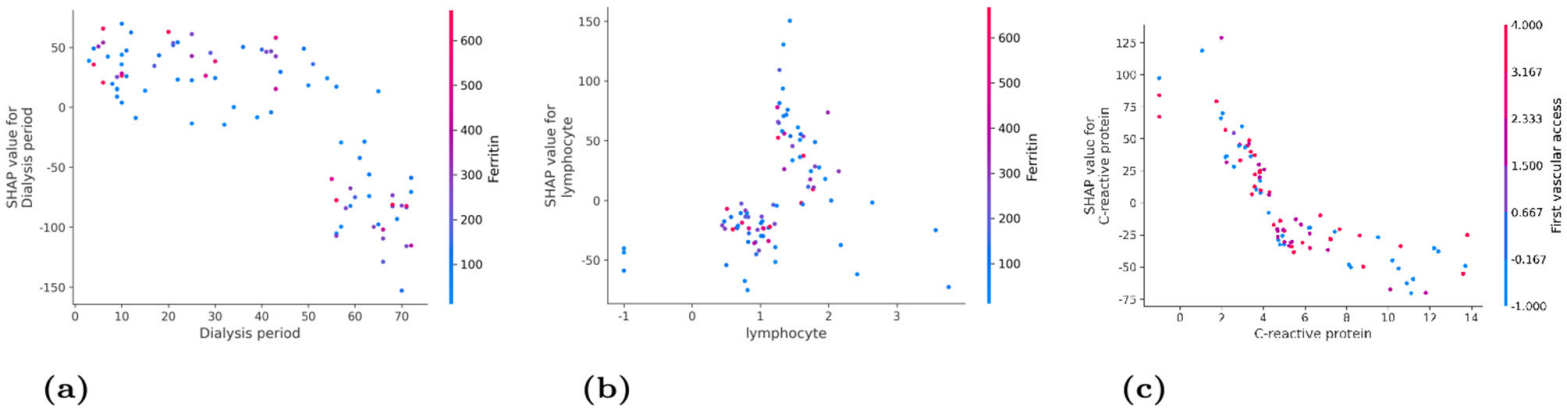
How the three most important factors affect outcomes at different values. **(a)** Dialysis period. **(b)** Lymphocyte count. **(c)** C-reactive protein.

Figure 8 shows important patterns:

- **Dialysis period**: - Less than 20 months: generally good outcomes - 20–40 months: neutral effect - More than 60 months: increasingly bad outcomes
- **Lymphocyte count**: - Below 1.0: bad outcomes (weak immune system) - 1.0–2.0: varies a lot (depends on other factors) - Above 2.0: generally better outcomes
- **C-reactive protein**: - Below 4 mg/L: normal, no negative effect - Above 4 mg/L: increasingly bad as inflammation gets worse

#### 3.5.5 How the model makes decisions

**Figure 9:**
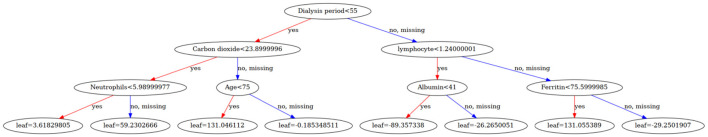
Decision tree 1 showing how the model makes predictions. Each tree asks yes/no questions about patient factors.

**Figure 10:**
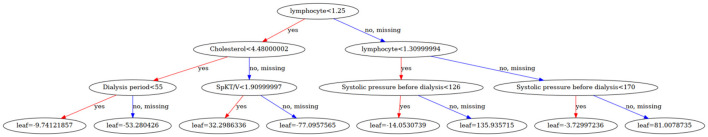
Decision tree 2 showing how the model makes predictions. Each tree asks yes/no questions about patient factors.

**Figure 11:**
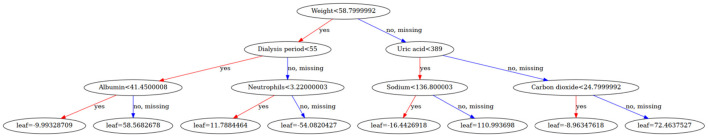
Decision tree 3 showing how the model makes predictions. Each tree asks yes/no questions about patient factors.

Figures 9–11 show the decision process.

- **Tree 1**: First checks dialysis period (split at 55 months)
- **Tree 2**: Focuses on lymphocyte count (split at 1.25)
- **Tree 3**: Looks at patient weight (split at 58.8 kg)

Each tree contributes to the final prediction by asking different questions about the patient.

#### 3.5.6 What this means for patient care

Our analysis reveals important insights for doctors and patients:

1. **Early intervention matters**: Patients on dialysis less than 20 months have better outcomes, suggesting the importance of optimizing care early.
2. **Monitor key indicators**: - Keep inflammation (CRP) below 4 mg/L - Maintain healthy lymphocyte counts (above 1.0) - Ensure good nutrition (albumin levels)
3. **Personalized care**: The complex patterns show that each patient is different—what works depends on their specific combination of factors.
4. **Focus on modifiable factors**: While we can't change how long someone has been on dialysis, we can work on: - Reducing inflammation - Improving nutrition - Maintaining healthy weight - Managing mineral balance

This analysis helps doctors identify which patients need extra attention and what specific areas to focus on for each individual patient.

## 4 Discussion

Patients undergoing hemodialysis have a high mortality rate from the peri-dialysis period to long-term maintenance. The median survival time has been reported to be approximately 5 years, with a gradual decline observed in different age (37). Therefore, precise individualized risk estimation and clinical decision support are needed. We developed a prognostic model to predict 3-year mortality in dialysis patients, employing a systematic comparison of multiple machine learning algorithms based on 32 readily available clinical features. The results show that XGBoost, AdaBoost, and CatBoost exhibit superior performance in clinical applications. Notably, AdaBoost demonstrates consistently stable and balanced performance across multiple optimization schemes, achieving the highest F1 score (0.684), reliable PR value (0.695), and AUC (0.894), making it highly applicable for similar mortality classification tasks. The model significantly reduces bias through its adaptive error correction mechanism. However, AdaBoost is less frequently used in previous hemodialysis prediction models.

In the SHAP importance analysis, albumin, CRP, and vascular access were revealed as the most important predictive indicators. Microinflammation is a fundamental pathological feature of dialysis patients and one of the most common pathways leading to death (38). The inflammatory immune status plays a core role in the prognosis of hemodialysis patients, which is consistent with previous studies (39). A recent large cohort study demonstrated that CRP levels are associated with a high risk of adverse renal outcomes (40). Inflammatory immune markers are closely related to infection risk, cardiovascular events, and dialysis adequacy, maintaining significant predictive value even within the normal reference range. This suggests the necessity for more standardized stratification and monitoring in clinical practice, at least for the quality control of CRP in hemodialysis. Hypoalbuminemia in hemodialysis patients represents malnutrition. Hypoalbuminemia is prone to cause hypotension during dialysis (41). Actively correcting hypoproteinemia in maintenance dialysis patients is a clinical consensus. To date, there is still widespread controversy over the best type of vascular access. Generally, it is believed that the risk of life-threatening complications is higher with the use of dialysis catheters compared to arteriovenous (AV) access (42). All patients with renal failure should have individualized strategies for renal replacement therapy and dialysis access. SHAP values provide feedback on both positive and negative features. In this study, we attempted to use XGBoost machine learning for visualization. As clinical decision-making requires early and precise individualized risk estimation, active clinical application is an urgent issue to be addressed. Additionally, this study attempted to use the method of multiple decision trees to simulate clinical scenarios, which helps to track the complete path from the root node to the leaf node and also provides paths specifically for missing values. To our knowledge, this is the first appearance of decision tree fragments in hemodialysis risk prediction.

Furthermore, the re-evaluation of clinical features after ablation further supports the prominent importance of inflammatory immune, hemodialysis indicators and nutritional indicators in the prognostic model. The hemodialysis indicators consist of dialysis adequacy, vascular access and dialysis months, which are directly noted factors in clinical practice. Nutritional indicators are often overlooked in clinical practice. Malnutrition is a common complication in maintenance hemodialysis patients and leads to a vicious cycle of high-risk mortality. However, there is not enough high-quality evidence to confirm that prolonged hemodialysis has a positive impact on nutritional parameters. How to identify patients with nutritional risk through screening tools is a key point for future clinical practice. However, blood pressure status showed a negative predictive effect. This counterintuitive finding may reflect time-dependent variation or reverse causality rather than true protective effects of elevated baseline blood pressure. Studies have found that intermittent hypotension during dialysis is a risk factor for death (43). In this cohort, the initial blood pressure status may only reflect vascular elasticity and volume at a single time point, ignoring the dynamic hemodynamic changes that occur throughout dialysis sessions. Incorporating time-series data with repeated blood pressure measurements and continuous monitoring of intradialytic blood pressure variability could better capture the true prognostic significance of hemodynamic status and refine risk prediction. In fact, the main purpose of the ablation experiment is not to compare model performance, but to determine key clinical factors and optimize clinical practice in a single-center context. The contribution of the remaining feature groups to the machine learning model is limited, merely indicating that the information value provided by this feature in a specific framework is insufficient.

However, in the current study, several limitations are worth considering: (1) The single-center retrospective study design may limit the generalizability of the research results, and prospective multicenter validation is needed to confirm the external applicability of these models. (2) The lack of independent validation in an external cohort is a significant limitation. (3) The relatively modest sample size (*n* = 538) and the imbalance of the class (approximately 23% mortality rate), although addressed through class weighting, can still limit the detection of rare clinical scenarios. (4) The scope of clinical characteristics needs to be expanded, especially in terms of primary diseases and imaging data. (5) The continuous learning mechanism of the model needs to be further refined. (6) Time series analysis has significant limitations. In summary, the multi-machine learning prediction system developed in this study provides new technical tools and clinical insights for the long-term prognosis assessment of hemodialysis patients. Future research will focus on multicenter external validation, address the limitations of existing prediction models, and promote the effective translation of prediction models into clinical practice.

## Data Availability

The data analyzed in this study is subject to the following licenses/restrictions: The dataset contains individual-level electronic health records that are protected under Chinese personal-information and medical-data regulations. Access is restricted to authorized researchers who obtain approval from the Affiliated Hospital of Guangdong Medical University's Ethics Committee and sign a data-use agreement ensuring confidentiality and compliance with applicable laws. Sharing with third parties requires additional ethical and legal review. Requests to access these datasets should be directed to Junfeng Hao ygzhjf85@gmail.com.
