# Supplementary material for: Use of machine learning models to predict mortality in dialysis patients
Source: Front Public Health. 2025 Dec 5;13:1683285. doi: 10.3389/fpubh.2025.1683285 (PMC12714995; doi:10.3389/fpubh.2025.1683285)
Supplement: Supplementary file 1 [file Data_Sheet_1.pdf]

Supplementary Materials

Supplementary Table S1: Summary of hyperparameter configurations used in our experiments.

Table 1: Summary of hyperparameter configurations used in our experiments.

Model	Hyperparameters
**LogisticRegression**	Penalty: {l1, l2, elasticnet, none}; C $\in$ {0.01, 0.1, 1, 10, 100}; Solver: {liblinear, saga, lbfgs}; Max iterations: 1000–20000; Class weight: {none, balanced}.
**Support Vector Machine (SVM)**	Kernel: {linear, rbf, poly, sigmoid}; C $\in$ {0.01, 0.1, 1, 10, 100, 1000}; γ $\in$ {scale, auto, 0.001, 0.01, 0.1, 1, 10}; Degree (poly): 2–5; Class weight: {none, balanced}.
**Random Forest (RF)**	n_estimators $\in$ {50, 100, 200, 300}; Max depth: {none, 10, 20, 30}; Min samples split: {2, 5, 10}; Min samples leaf: {1, 2, 4}; Feature selection: {sqrt, log2, none, 0.7}; Bootstrap: {true, false}.
**XGBoost**	n_estimators $\in$ {50, 100, 200, 300}; Max depth: {3–8}; Learning rate: {0.05–0.2}; Min child weight: {1, 3, 5}; Subsample: {0.8–1.0}; Colsample bytree: {0.8, 0.9}; Regularization: $\alpha \in$ {0, 0.1, 0.5}, $\lambda \in$ {1.0, 1.5}.
**Neural Network (MLP)**	Layers: 2–4; Hidden units per layer: 64–256; Dropout: 0.2–0.5; Optimizer: Adam (lr=$10^{-3}); Batch size: 16–32; Early stopping patience: 20.
**LSTM**	Recurrent layers: 1–2; Hidden dimension: 64–128; Fully connected layers for output; Dropout: 0.2–0.5; Optimizer: Adam (lr=$10^{-3}).
**Transformer Classifier**	Hidden dimension: d_model = 128; Attention heads: 8; Layers: 2; Feed-forward dimension: 512; Dropout: 0.1; Optimizer: Adam (lr=$10^{-3}) with cosine annealing; Batch size: 4; Early stopping patience: 300 epochs.

Supplementary Table S2: Software environment and key functionalities used in this study

Table 2: Software environment and key functionalities used in this study

Library/Tool	Version	Purpose
AutoGluon	v1.x	Automated machine learning (AutoML), model ensembling, leaderboard generation.
scikit-learn	v1.x	Classical ML models (LR, SVM, RF, AdaBoost, etc.), preprocessing, evaluation metrics.
XGBoost	v1.x	Gradient boosting decision trees and feature importance analysis.
LightGBM	v3.x	Efficient gradient boosting for tabular data.
CatBoost	v1.x	Gradient boosting with categorical feature support.
PyTorch	v2.x	Implementation of deep learning models (NN, LSTM, Transformer).
pandas	v1.x	Data loading, cleaning, and manipulation.
NumPy	v1.x	Numerical computations and array operations.
Matplotlib	v3.x	Visualization (ROC/PR curves, confusion matrices, SHAP plots).
Seaborn	v0.x	Heatmaps and statistical data visualization.
SHAP	v0.x	Model interpretability (feature importance, summary plots, dependence plots).

Table 3: Patient demographics and baseline characteristics

**Characteristic**	**Overall**<br>**N = 538**	**Survivors**<br>**N = 415**	**Non-survivors**<br>**N = 123**	**p-value**
24 hour urine-output, Mean ± SD	467 ± 479	486 ± 487	404 ± 450	0.084
Dialysis period, Mean ± SD	33 ± 24	34 ± 24	30 ± 23	0.073
Weight, Mean ± SD	61 ± 11	60 ± 11	62 ± 11	0.059
Systolic pressure before dialysis, Mean ± SD	149 ± 22	149 ± 22	147 ± 23	0.264
Diastolic blood pressure before dialysis, Mean ± SD	78 ± 14	79 ± 14	76 ± 15	0.032
Neutrophils, Mean ± SD	4.59 ± 1.89	4.55 ± 1.94	4.74 ± 1.70	0.296
Lymphocyte, Mean ± SD	1.20 ± 0.51	1.20 ± 0.48	1.16 ± 0.58	0.465
Platelet, Mean ± SD	210 ± 74	214 ± 76	199 ± 66	0.033
Hemoglobin, Mean ± SD	103 ± 19	104 ± 19	100 ± 19	0.021
C-reactive protein, Mean ± SD	11 ± 26	10 ± 27	12 ± 20	0.413
Creatinine, Mean ± SD	865 ± 273	890 ± 277	777 ± 239	< 0.001
Urea nitrogen, Mean ± SD	27 ± 8	27 ± 8	26 ± 8	0.215
Uric acid, Mean ± SD	448 ± 113	447 ± 108	449 ± 128	0.854
Sodium, Mean ± SD	137.92 ± 2.72	138.05 ± 2.58	137.48 ± 3.14	0.072
Potassium, Mean ± SD	4.80 ± 0.70	4.82 ± 0.67	4.73 ± 0.80	0.263
Phosphorus, Mean ± SD	2.08 ± 0.56	2.10 ± 0.56	2.02 ± 0.57	0.196
Albumin, Mean ± SD	38.9 ± 4.3	39.4 ± 4.4	37.2 ± 3.6	< 0.001
Cholesterol, Mean ± SD	4.03 ± 1.06	4.03 ± 1.04	4.01 ± 1.14	0.882
Triglyceride, Mean ± SD	1.65 ± 1.12	1.72 ± 1.17	1.41 ± 0.87	0.002
Calcium, Mean ± SD	2.22 ± 0.23	2.22 ± 0.23	2.21 ± 0.20	0.515
Blood sugar, Mean ± SD	7.60 ± 3.04	7.57 ± 3.00	7.70 ± 3.18	0.692
Carbondioxide, Mean ± SD	19.1 ± 3.1	19.1 ± 3.2	19.1 ± 3.0	0.950
Ferritin, Mean ± SD	228 ± 209	221 ± 204	250 ± 224	0.201
β-microglobulin, Mean ± SD	16.7 ± 4.0	16.7 ± 3.9	17.0 ± 4.1	0.470
Parathyroid hormone, Mean ± SD	532 ± 486	536 ± 474	519 ± 529	0.751
SpKT/V, Mean ± SD	1.45 ± 0.33	1.45 ± 0.32	1.43 ± 0.35	0.630
Sex, n (%)				0.049
Male	331 (61.5%)	246 (59.3%)	85 (69.1%)	
Women	207 (38.5%)	169 (40.7%)	38 (30.9%)	
Hypertension, n (%)				0.303
No	37 (6.9%)	26 (6.3%)	11 (8.9%)	
Yes	501 (93.1%)	389 (93.7%)	112 (91.1%)	
Diabetes, n (%)				0.021
No	353 (65.6%)	283 (68.2%)	70 (56.9%)	
Yes	185 (34.4%)	132 (31.8%)	53 (43.1%)	
First vascular access, n (%)				0.182
Arteriovenous fistula	472 (87.7%)	358 (86.3%)	114 (92.7%)	
Temporary catheter	27 (5.0%)	23 (5.5%)	4 (3.3%)	
Long-term catheter	28 (5.2%)	23 (5.5%)	5 (4.1%)	
Arteriovenous graft	11 (2.0%)	11 (2.7%)	0 (0.0%)	
Cardiovascular diseases, n (%)				0.004
No	415 (77.1%)	332 (80.0%)	83 (67.5%)	
Yes	123 (22.9%)	83 (20.0%)	40 (32.5%)	
